# Computational methods for the ab initio identification of novel microRNA in plants: a systematic review

**DOI:** 10.7717/peerj-cs.233

**Published:** 2019-11-11

**Authors:** Buwani Manuweera, Gillian Reynolds, Indika Kahanda

**Affiliations:** 1 Gianforte School of Computing, Montana State University, Bozeman, MT, United States of America; 2 Department of Plant Sciences and Plant Pathology, Montana State University, Bozeman, MT, United States of America

**Keywords:** ab initio, microRNA, Plant, Machine learning, Systematic review

## Abstract

**Background:**

MicroRNAs (miRNAs) play a vital role as post-transcriptional regulators in gene expression. Experimental determination of miRNA sequence and structure is both expensive and time consuming. The next-generation sequencing revolution, which facilitated the rapid accumulation of biological data has brought biology into the “big data” domain. As such, developing computational methods to predict miRNAs has become an active area of inter-disciplinary research.

**Objective:**

The objective of this systematic review is to focus on the developments of ab initio plant miRNA identification methods over the last decade.

**Data sources:**

Five databases were searched for relevant articles, according to a well-defined review protocol.

**Study selection:**

The search results were further filtered using the selection criteria that only included studies on novel plant miRNA identification using machine learning.

**Data extraction:**

Relevant data from each study were extracted in order to carry out an analysis on their methodologies and findings.

**Results:**

Results depict that in the last decade, there were 20 articles published on novel miRNA identification methods in plants of which only 11 of them were primarily focused on plant microRNA identification. Our findings suggest a need for more stringent plant-focused miRNA identification studies.

**Conclusion:**

Overall, the study accuracies are of a satisfactory level, although they may generate a considerable number of false negatives. In future, attention must be paid to the biological plausibility of computationally identified miRNAs to prevent further propagation of biologically questionable miRNA sequences.

## Introduction

microRNAs (miRNAs) are a large family of small (approx. 20–25 nucleotides) single-stranded RNAs, involved in post-transcriptional gene regulation through the cleavage and/or inhibition of target mRNAs (Rogers & Chen, 2013; Voinnet, 2009). Despite being found throughout the eukaryotic kingdom, plant microRNAs differ from their metazoan counterparts in a number of ways, including their genomic loci (regions in which their genes can be found i.e., introns, UTRs, etc.), biogenesis, length, methods of target recognition and number of targets per miRNA molecule (Axtell, Westholm & Lai, 2011; Moran et al., 2017). Computationally, plant and animal miRNAs can be differentiated through several distinguishing characteristics such as helix number, stack number, length of pre-miRNA and minimum free energy (Zhu et al., 2016). Indeed, it is currently uncertain if plant and animal microRNAs share a common origin or if they evolved independently in both lineages (Axtell, Westholm & Lai, 2011; Moran et al., 2017; Zhang et al., 2018).

Despite the uncertainty regarding their origin, it has never been more important for the focused characterization of plant microRNAs. Production levels for many of the world’s crops are under threat from increases in global temperatures, changing patterns of rainfall and extreme weather events such as droughts, heatwaves and heavy rainfall (Mall, Gupta & Sonkar, 2017). A meta-analysis of over 1,700 simulations for wheat, rice and maize has indicated that an increase of just 2 degrees will cause losses in aggregate production (Challinor et al., 2014). Between 2030–2052, the Intergovernmental Panel on Climate Change (IPCC) reports with high confidence that global temperature increases of 1.5 degrees is likely to become a reality if current rates of temperature changes are maintained (Hoegh Guldberg et al., 2018). Although this will result in smaller net reductions for maize, rice, wheat and potentially other cereal crops than would be observed with a 2 degree rise, the risk to global food security and economics is not to be overlooked, especially regarding staple crops such as wheat, that are required to increase in production levels to meet projected increases in global demands (Liu et al., 2016; Ray et al., 2013; Hoegh Guldberg et al., 2018; Challinor et al., 2014).

miRNAs are known to be involved in several important stress-response pathways including drought, heat and salinity. For example, in the model plant *Arabidopsis thaliana*, upregulation of miR389 is critical for thermotolerance (Guan et al., 2013), downregulation of miR169 is observed in drought-tolerant varieties and overexpression of osa-MIR396c inferred increased salt and alkali tolerance (Gao et al., 2010). However, it has become clear that plant species show remarkable variety in the relationship between miRNAs and their role in stress tolerance. For example, osa-MIR396c in rice (*Oryza sativa*) showed the same response as *A. thaliana* in increased salinity and alkaline environments (Gao et al., 2010). However, for other miRNAs such as miR169 the relationship between their expression and drought tolerance appears to vary between species. In *A. thaliana* and the model legume *Medicago truncatula*, miR169 is down-regulated in response to drought (Li et al., 2008; Trindade et al., 2010; Sunkar, Li & Jagadeeswaran, 2012). Contrastingly, in rice and tomato (*Solanum lycopersicum cv. Ailsa Craig*), drought stress led to the up-regulation of miR-169 (Zhao et al., 2007; Zhang et al., 2011). Additionally (Zhou et al., 2010) identified a further 9 miRNAs that showed opposite expression patterns in *A.thaliana* in response to drought stress. The observed interspecies variation in miRNA activity in response to stressful stimuli demonstrates that there is a need for the discovery and functional characterization of miRNAs for each species of plant of interest.

Thanks to advancements in next-generation sequencing (NGS) technology and interdisciplinary collaborations, the rapid identification of species-specific plant miRNAs and their expressions in response to stimuli is now possible (Liu et al., 2012; Unamba, Nag & Sharma, 2015; Hu, Lan & Miller, 2017). NGS is both high throughput and highly accurate, facilitating the identification of sequence variations and novel miRNAs (Hu, Lan & Miller, 2017). However, many computational methods such as those described in (Evers et al., 2015; An et al., 2014; Hackenberg, Rodriguez-Ezpeleta & Aransay, 2011) only allow for homology-based identification of miRNAs. This means the tools are not able to take full advantage of the available information in the sequencing data, such as novel miRNA identification. As such, numerous ab initio methods have been developed to facilitate the discovery of novel miRNAs. However, caution is being urged when interpreting the results of such computational inferences of biological data (Taylor et al., 2017; Taylor et al., 2014). The generation of computational tools to identify miRNA sequences requires biological assumptions to underpin the methods and, as with all new areas of research, these assumptions change with new evidence over time (Ambros et al., 2003; Meyers et al., 2008; Axtell & Meyers, 2018).

This systematic review surveys the computational methods that facilitate the ab initio identification of plant miRNAs over the last decade (2008–2018). It seeks answers to five research questions that aim to elucidate the developments, reliability and validity of the methods used, and considers potential opportunities for future developments in the computational identification of miRNAs.

## Methodology

This systematic review focuses on the literature that was published between 2008 and 2018. This time range was considered to collect and analyze the recent methodologies developed on ab initio plant miRNA identification.

The following sections contain the steps of the review protocol: research questions, search strategy, selection criteria, data extraction and quality assessment.

### Research questions

This review is intended to answer the following research questions:

(Q1) How many methods were developed during the past decade?
(Q2) What kind of machine learning algorithms and features were used? Which models/features performed well?
(Q3) How accurate and reliable are the developed models?
(Q4) What kind of computational and/or experimental validation methods were used? How appropriate are those validation methods?
(Q5) What are knowledge gaps, open problems and/or opportunities?

### Search strategy

The search strategy was used to identify plant miRNA prediction methods developed between 2008 and 2018 in databases of IEEE Xplore (https://ieeexplore.ieee.org/Xplore/home.jsp), Science Direct (https://www.sciencedirect.com/), PubMed (https://www.ncbi.nlm.nih.gov/pubmed?otool=msubolib), Web of Science (http://www.webofknowledge.com/) and Google Scholar (https://scholar.google.com/). The following terms were used for the literature searches: “novel miRNA identification in plants” (including variations of the word “identification” such as “prediction” and “discovery”) and “computational method”. They were used as queries as shown below.

(novel miRNA identification in plants) **AND** (computational method)

These search terms were utilized to narrow down the large number of mostly-irrelevant retrieved articles from databases such as Science Direct and Google Scholar, into mostly relevant articles.

### Selection criteria

The selection criteria used for the review is shown in Table 1.

**Table 1:** Article selection criteria.

**Inclusion criteria**	**Exclusion criteria**
Studies that use machine learning algorithms	Studies that only use sequence homology
Studies that solely use plants or include plant data	Studies that use animal or unspecified species datasets
Published journal articles or conference proceedings	Literature reviews/surveys on the subject and unpublished articles

The review process began with a study search procedure. From the initial search results to the final list of primary studies, the procedure was performed as follows.

1. The article search was carried out using the aforementioned search strategy mentioned above. A total of 2,738 search results were found from all of the databases. That is considering only 300 search results from Google Scholar as it gave over 18,000 results per search term. In order to narrow-down from 18,000 Google Scholar results, we restricted the output to the first ten pages of the search. This resulted in 300 articles that are most relevant to the query.
  • IEEE Xplore: 116
  • Science Direct: 2140
  • PubMed: 116
  • Web of Science: 66
  • Google Scholar: 300
2. Out of the search results from the databases, articles were first filtered by assessing the title’s relevance. If deemed relevant to the subject, it was included in the initial list.
3. Secondly, the abstracts were assessed for relevance. This resulted in 41 articles.
4. Finally, the selection criteria (see Table 1) were applied on the remaining 41 articles and 20 articles were retained as the final list (referred to as the primary list).

### Data extraction

Table 2 outlines the criteria used for data extraction from the 20 primary studies. Data and general information from each article were extracted to enable the five research questions to be addressed (see Research Questions).

**Table 2:** Data extraction form.

**Search focus**	**Data item**	**Description**
General	Article Details	Title, Authors, Published year and publication venue
	Article Type	Journal article or conference proceedings
	Study Description	Introduction of the study
Q1	Data	Plant data only methods and methods including plant data
Q2	Datasets	Dataset source, positive and negative example datasets, and species
	Features	Types of features used
	Machine Learning Algorithms	Type of machine learning algorithm used for classification
	Feature Selection	Methods used to select/extract features for the model
Q3	Performance Metrics	Accuracy values and other performance measurements
Q4	Validation Methods	Cross-validation and Experimental validation methods
Q5	Future Work	Suggested future work in Conclusion section and other aspects that are not being addressed

### Quality assessment

The study quality assessment was performed on all 20 primary studies and was based on six questions as detailed:

(QA1) Are all the considered data being used for the model (without sample selection)? A “sample” refers to a single miRNA sequence considered for the experiments. In machine learning, they are also referred to as an example or an instance of data.
(QA2) Do they mention any information about the negative dataset used? A typical machine learning model require positive and negative examples, which are sequences labeled as miRNAs or none-miRNAs, respectively. This question refers to any information about the negative dataset such as what kind of sequences were considered as negatives and how many examples were considered.
(QA3) Are there any feature selection methods considered in each method? Rather than using all the features gathered, did the study use a feature selection method to select a subset of most effective features for model development.
(QA4) Do they conduct any experimental validation of their findings? Did the study use validation methods to experimentally validate the findings (miRNA predictions) output from their machine learning models.
(QA5) Are the results of the performance evaluation quantified? Did the study present their results using a typical performance measure such as accuracy used in machine learning.
(QA6) Is the study focused only on plant miRNA identification? Did the study solely use plant miRNA sequences for developing the prediction model or have they considered a mixture of plant and animal miRNAs.

## Results

**Figure 1:**
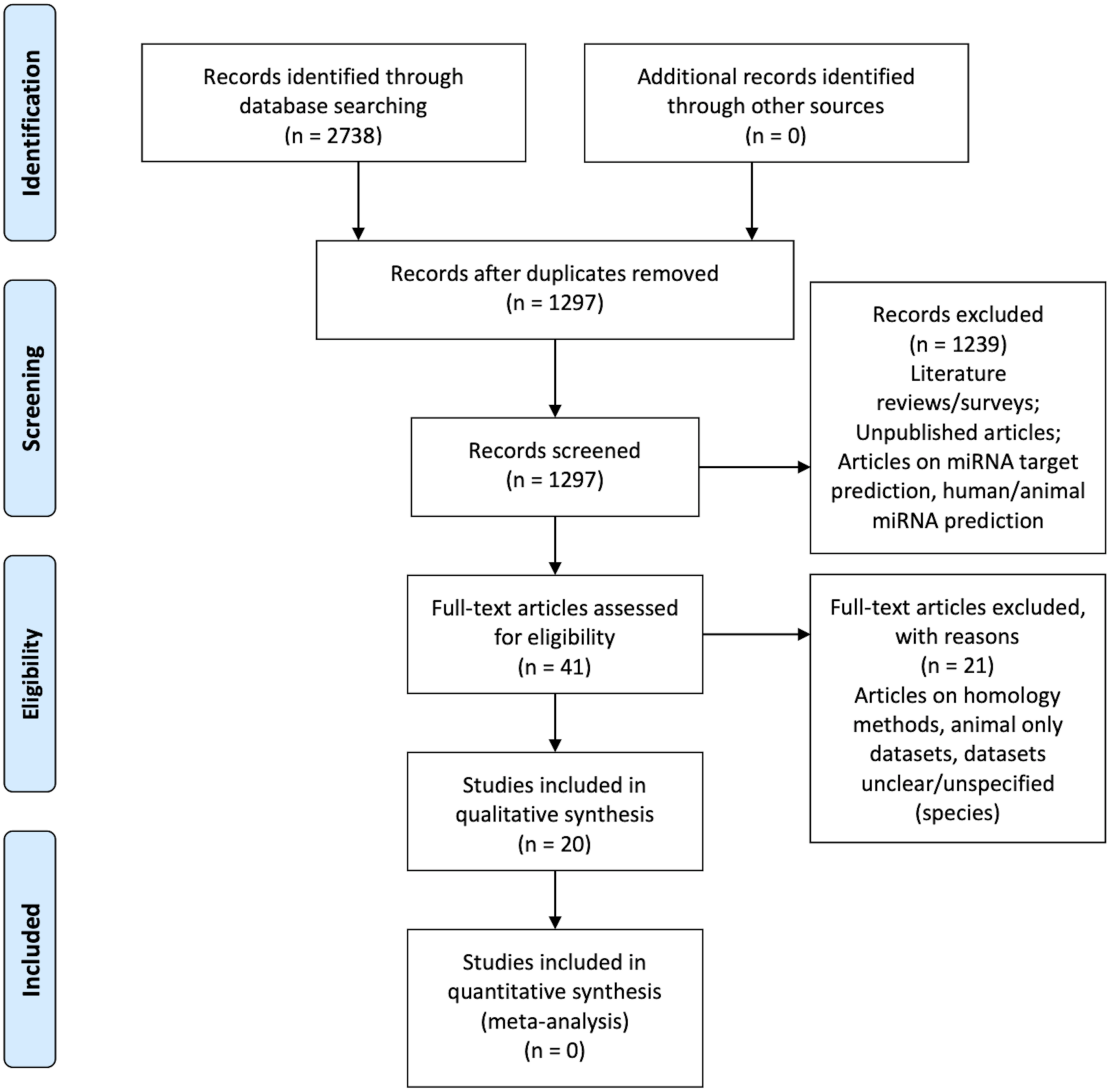
PRISMA Flow Diagram (Liberati et al., 2009).

Figure 1 is the flow diagram depicting the study selection process with the numbers described in the methodology (Liberati et al., 2009).

Table 3 illustrates the results of the quality assessment process. None of the articles answered “Yes” to all the six questions. Zou et al. (2014) does not satisfy any quality assessment category, but it is still considered for the systematic review in order to analyze their methodology.

**Table 3:** Quality assessment results.

**Reference**	**QA1**	**QA2**	**QA3**	**QA4**	**QA5**	**QA6**
Tseng et al. (2018)	No	Yes	Yes	Yes	Yes	Yes
Yousef et al. (2016)	Yes	Yes	Yes	No	Yes	Yes
Yousef, Allmer & Khalifa (2015)	Yes	Yes	Yes	No	Yes	Yes
Breakfield et al. (2012)	No	Yes	No	Yes	Yes	Yes
Douglass et al. (2016)	No	Yes	No	Yes	Yes	Yes
Sunkar et al. (2008)	No	Yes	Yes	Yes	No	Yes
Abu-halaweh & Harrison (2010)	Yes	Yes	Yes	No	Yes	No
Guan et al. (2011)	Yes	Yes	Yes	No	Yes	No
Meng et al. (2014)	No	Yes	Yes	No	Yes	Yes
Williams, Eyles & Weiller (2012)	No	Yes	Yes	No	Yes	Yes
Xuan et al. (2011)	No	Yes	Yes	No	Yes	Yes
Yao et al. (2016)	Yes	Yes	No	No	Yes	Yes
Koh & Kim (2017)	Yes	Yes	No	No	Yes	No
Silla, De O Camargo-Brunetto & Binneck (2010)	No	Yes	No	No	Yes	Yes
Wu et al. (2011)	No	Yes	Yes	No	Yes	No
Zhong et al. (2015)	Yes	Yes	No	No	Yes	No
Kadri, Hinman & Benos (2009)	No	Yes	No	No	Yes	No
Vitsios et al. (2017)	Yes	No	No	No	Yes	No
Xiao et al. (2011)	No	Yes	No	No	Yes	No
Zou et al. (2014)	No	No	No	No	No	No

Tables 4 and 5 shows the information collected from the primary studies during the data extraction process. Table S2 shows the publication venues of the primary articles. According to the table, BMC Bioinformatics journal has the most number of articles selected.

**Table 4:** Data extraction results.

**Primary study**	**Article type**	**Data**	**Dataset source**	**Number of species used**	**Negative datasets**	**Feature selection methods**
Xuan et al. (2011)	J	P	miRBase 14, Phytozome 6 database	29	Protein coding region of A.thaliana and G.max genomes	Considering information gain and feature redundancy
Yousef et al. (2016)	J	P	miRBase 20, 21	5 in Brassicaceae and training data from Xuan et al. (2011)	Samples from Xuan et al. (2011)	Using SVM-RFE (Recursive feature elimination) implemented in WEKA, selected top 60 ranked features.
Silla, De O Camargo-Brunetto & Binneck (2010)	C	P	Plant MicroRNA Database, deepBase, Phytozome	131 Glycine max, 199 Athaliana, 100 Medicago truncatula	175 Arabidopsis thaliana snoRNA sequences from deepBase2 and 225 RNA sequences randomly generated	N/A
Meng et al. (2014)	J	P	miRBase 19		From coding regions of 3 species	Using Back SVM-RFE, 47/152 features were selected
Breakfield et al. (2012)	J	P	miRBase 16, NCBI Sequence Read Archive	Arabidopsis	From intergenic or intronic genomic locations	N/A
Douglass et al. (2016)	J	P	miRBase 21, Gene Expression Omnibus (GEO)	4	smRNA sequences remaining after known miRNA filtering	N/A
Yao et al. (2016)	J	P	miRBase 21, EnsemblPlants database v18	9	From coding region of 5 species	Selected subsets of features (based on types of features) to check the impact of those features
Tseng et al. (2018)	J	P	miRBase 21, Gene Expression Omnibus, TAIR, RGAP	Arabidopsis and Rice		Tested with different combinations of features (based on type)
Williams, Eyles & Weiller (2012)	J	P	miRBase 18, TIGR Plant Transcript Assemblies	18	From Expressed Sequence Tags (EST) of 18 species	N/A
Sunkar et al. (2008)	J	P	miRBase 9, TIGR Rice Genome Annotation Database	Rice	Rice coding sequences from TIGR	Wrapper-based method. Using weights from SVM
Yousef, Allmer & Khalifa (2015)	J	P	miRBase 20, 21	8	From Xuan et al. (2011)	Using SVM-RFE (Recursive feature elemination) implemented in WEKA, selected top 60 ranked features.
Xiao et al. (2011)	J	Eval: P+V	miRBase 14	All miRBase 14	From previous work (Human data)	N/A
Koh & Kim (2017)	J	A+P	miRBase 21	miRBase21 excluding virus	Pseudo hairpins form microPred	N/A
Wu et al. (2011)	J	A+P	miRBase 13	All miRBase 13	Random start sequences; identical to real miRNA but start position is shifted by 5nt	Tested for the 10 highest raninking features
Zou et al. (2014)	J	A+P	miRBase 19	All miRBase 19		Tested on different feature sets
Zhong et al. (2015)	J	A+P	Previous studies (miRBase 12, 14, 17)	From previous studies	Previous methods	N/A
Guan et al. (2011)	J	A+P	miRBAse 12		From protien coding regions (from previous studies)	N/A
Vitsios et al. (2017)	J	A+P	miRBase	15		N/A
Abu-halaweh & Harrison (2010)	C	A+P	previous work (Rfam 5 etc.)	12	Human coding regions	FDT integrates two measures, Classification Ambiguity and Fuzzy Information Gain to identify the set of the most significant features
Kadri, Hinman & Benos (2009)	J	Test set: P	microRNA registry v10.1, UCSC genome browser	2	Coding regions and random genomic segments from genome obtained by UCSC genome browser	N/A

**Notes.**

J Journal C Conference proceeding P Plant A Animal V Virus N/A Feature selection not used

**Table 5:** Data extraction results (2).

Primary study	Input	Types of features				Types of ML models		Predicted output	Key results							Experimental validation
		Sequence	Structural	Thermodynamic/ Stability	Other	Discriminative	Probabilistic		Precision	Recall	F1-score	Specificity	Geometric mean	Accuracy	AUC	
Xuan et al. (2011)	pre-miRNA	17	64 triplet	34		SVM - RBF Kernel		pre-miRNA		91.93		97.84	94.84	94.39		
Yousef et al. (2016)	pre-miRNA	existing; motif features	existing	existing		SVM - RBF Kernel		pre-miRNA		98.8		100		99.48	0.994	
Silla, De O Camargo-Brunetto & Binneck (2010)	pre-miRNA	17		12		SVM - RBF Kernel		pre-miRNA		89		95		92		
Meng et al. (2014)	pre-miRNA and mature miRNA	20	96	29		SVM		pre-miRNA and mature miRNA		95.5		98.82	97.16	97.16		
Breakfield et al. (2012)	small RNA				15 including all types		Naïve Baye’s	Mature miRNA vs nc-RNA		91.7		99.9				RT-PCR etc.
Douglass et al. (2016)	small RNA						Naïve Baye’s	mature miRNA							0.998	RT-PCR
Yao et al. (2016)	pre-miRNA				Including all types	SVM - RBF Kernel		pre-miRNA		92.61		98.88		96.56	0.9,885	
Tseng et al. (2018)	small RNA	1	183	1		SVM		mature miRNA	95.22	98.15		95.07		96.61		RT-PCR
Williams, Eyles & Weiller (2012)	mature miRNA	22	4	3		Decision Tree		mature miRNA		84.08		98.53				
Sunkar et al. (2008)	small RNA	4 to 9-mer seq. motifs				SVM - Linear		mature miRNA								Northern analysis
Yousef, Allmer & Khalifa (2015)	pre-miRNA	n-grams, motifs				SVM and K-means		mature miRNA						91.4		
Xiao et al. (2011)	pre-miRNA		24 network features of stem-loop			Random Forest		pre-miRNA		87.3		91.1		97.6	0.956	
Koh & Kim (2017)	pre-miRNA	17		12		SVM - RBF Kernal		pre-miRNA		96		94.68				
Wu et al. (2011)	pri-miRNA	6,5,5 mature, pre, pri-mirna	9,5,5 mature, pre, pri-mirna	1,1,1 mature, pre, pri-mirna	30 other features	SVM		mature miRNA regions						80		
Zou et al. (2014)	mature miRNA	4,096	32 triplet			Random Forest		mature miRNA and their family								
Zhong et al. (2015)	pre-miRNA	81	49	9		SVM		pre-miRNA		93.37		97.91	95.61			
Guan et al. (2011)	pre-miRNA				misc. covering all types	ADABoost		pre-miRNA and mature miRNA		94.32		97.11	96	97.54		
Vitsios et al. (2017)	mature miRNA				33 covering all types	Random Forest		Mature miRNA						71.4-71.8		
Abu-halaweh & Harrison (2010)	pre-miRNA				Including all types	Fuzzy Decision Tree		pre-miRNA		91.5		94.7		94.2		
Kadri, Hinman & Benos (2009)	pre-miRNA				4 parameters		Hierarchical HMM	pre-miRNA		84		88				

**Notes.**

F1 Score 2*(Precision*Recall)/(Precision+Recall) Geometric Mean Sensitivity*Specificity

The answers to all the research questions are being presented below based on the primary studies selected.

(Q1) How many methods were developed during the past decade?

The primary list of articles consisted of 20 studies which were focused on the problem of novel plant miRNA identification. Of these, 11 studies were focused solely on plant miRNA identification. The remaining studies focused on both plant and animal miRNA identification, with plant datasets either used to train the machine learning models or used only to test the model (after training with non-plant datasets).

The plant-focused studies used datasets from several different species. Meng et al. (2014) considered all the plant datasets available in miRBase (a miRNA database) by (Kozomara & Griffiths-Jones, 2014). Breakfield et al., 2012; Silla, De O Camargo-Brunetto & Binneck, 2010 and Sunkar et al., 2008, each worked on one specific plant species (Arabidopsis, soybean and rice respectively). Therefore, they used only that plant species or included a few additional species to the dataset. As there is not an abundance of species-specific miRNA data available, most studies used a combination of plant species data.

The primary list contains nine studies that used both plant and animal datasets. These studies used the same features for both kingdoms miRNA identification. This might be due to the lack of data in plants. Therefore, researchers tend to combine animal datasets in order to get a larger dataset, and they consider the same features. This results in a number of tools that are for both animals and plants that do not consider the differences between their miRNAs.

**Figure 2:**
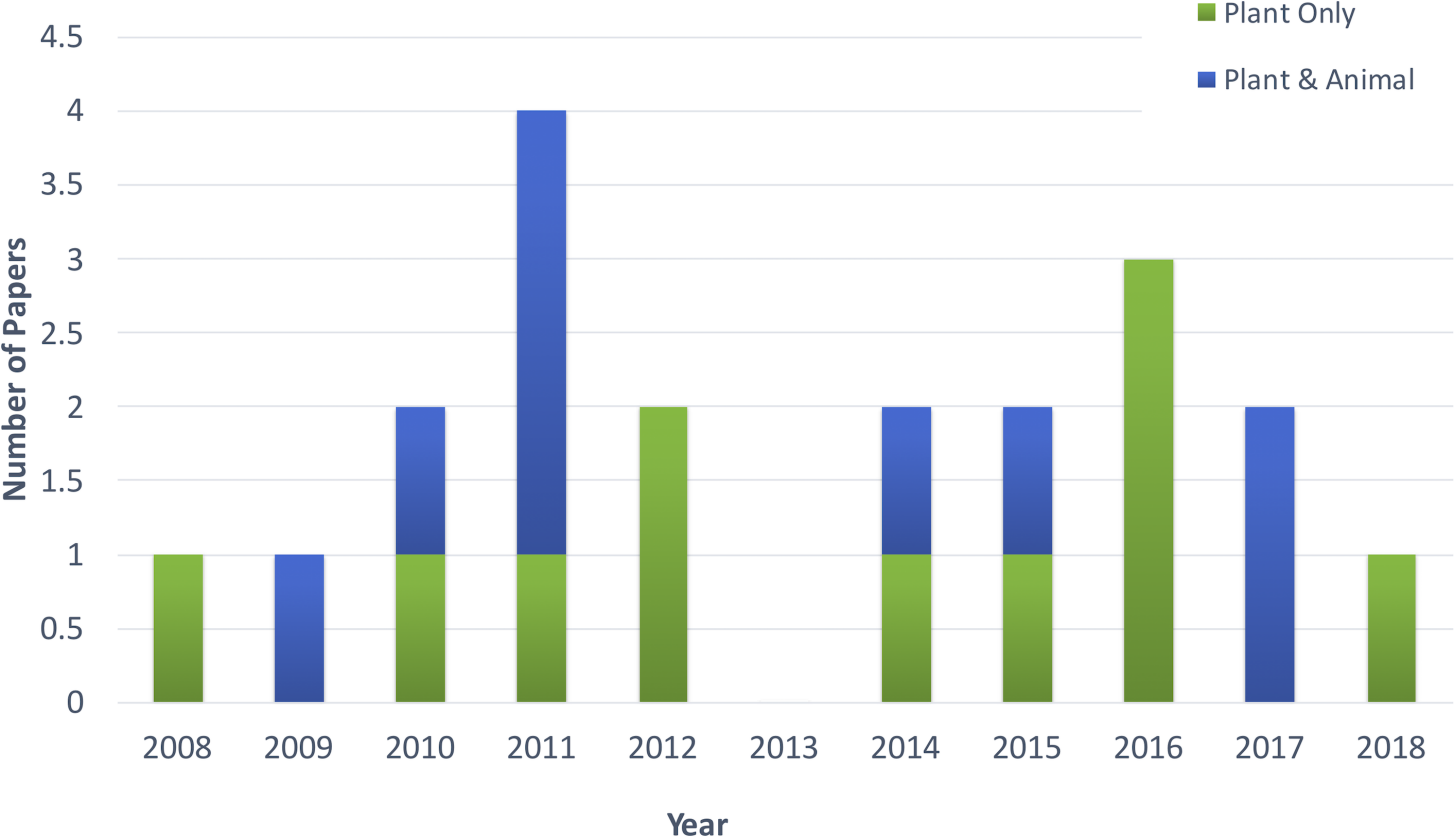
Distribution of publications in the past decade. .

**Figure 3:**
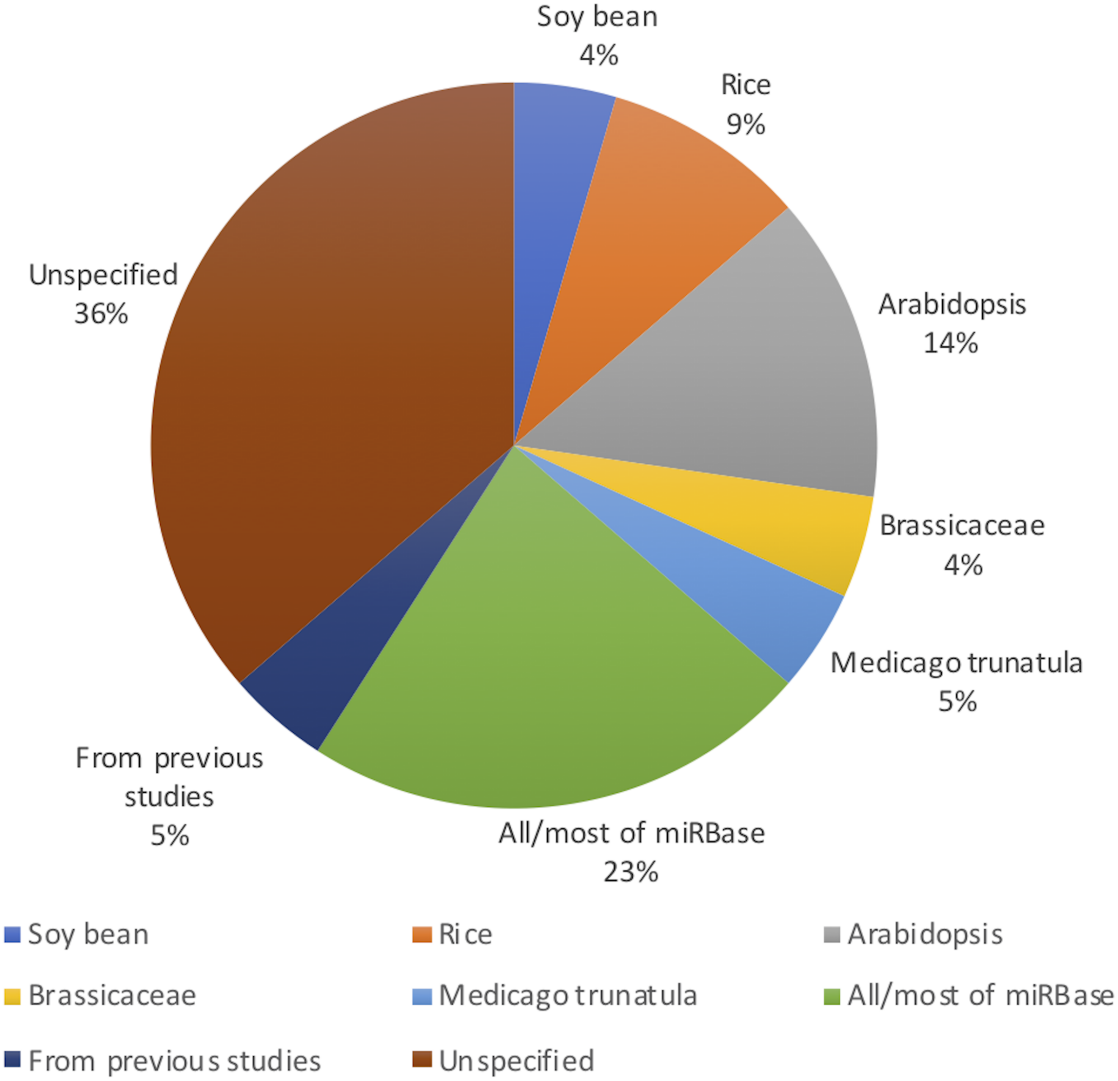
Plant species used (even though Arabidopsis belongs to the Brassicaceae family, it has been used in significant amount of work as it is a model plant; therefore, it has been added to the figure separate from Brassicaceae).

Figure 2 shows the distribution of article publication on the subject in the past decade. Most plant only publications occurred in 2016 and 2013, no publication was published on novel plant miRNA identification. Figure 3 shows the distribution of specific plant species used in the primary studies.

All the studies used both positive and negative datasets in their methods. Whilst plant miRNA data was used for the positive set, a range of data was used for the negative set, ensuring they were free of real miRNA sequences. Nine studies used protein coding regions to collect pseudo miRNAs for their negative dataset. As almost all reported miRNAs are found in the non-coding regions of the genome, these sequences are assumed as pseudo miRNA data (Xuan et al., 2011). Guan et al. (2011), Koh & Kim (2017), Xiao et al. (2011), Yousef, Allmer & Khalifa (2015) and Yousef et al. (2016) used the negative datasets from previous studies which were already available. Yousef, Allmer & Khalifa (2015) discusses a one-class classifier for plant miRNAs where they only used the positive data set. However, for the comparison with a binary classifier, they needed a negative dataset. The remaining studies either randomly generated negative datasets or used other non-coding RNAs such as small nucleolar RNA (snoRNA), transfer RNA (tRNA) etc.

(Q2) What kind of machine learning algorithms and features were used? Which models/features performed well?

Many of the studies used the same or similar sets of features consisting of sequence-based, structural and thermodynamic features. The studies use either the same set of features from previous studies or extend them by adding new features to enhance performance. The sequence-based features often consist of nucleotide/di-nucleotide frequencies, motifs, n-grams, GC content and sequence length among others.

The structural features primarily consist of features as described in Xue et al. (2005) and also minimum free energy (MFE) measures. Thermodynamic features include the structure entropy and enthalpy measures. The vast majority of studies utilize a combination of various structural and sequence-based features which may aid in increasing the chances of identifying a correct miRNA, despite their diversity within the plant kingdom.

Williams, Eyles & Weiller (2012) and Kadri, Hinman & Benos (2009) have used sliding windows of size ranging 300–500 nt (known plant pre-miRNA are below 300 nt according to Williams, Eyles & Weiller (2012) and for Kadri, Hinman & Benos (2009), most of the pre-miRNA were covered when the window size is 500 nt) to scan genome sequences for folding into hairpin structures and then collect structural features. Therefore, this range can be used for scanning the whole genome of a specific plant spices.

Plant precursor sequences have varying sizes of secondary structures but there is no unified technique reported for dealing with the issue. Williams, Eyles & Weiller (2012) select the size of the majority of pre-miRNA in miRBase (<300 nt). Kadri, Hinman & Benos (2009) use 50 nt minimum for selecting/ filtering pre-miRNAs. Xuan et al. (2011) considered different ranges of lengths to get the majority of sequence information. Wu et al. (2011) used 100 nt as the length of pre-miRNA. According to Meng et al. (2014), plant pre-miRNA can range from 53–938 nt. Therefore, many of the studies have used a window size that is being guided by this length range to select the set of pre-miRNA for their studies.

**Figure 4:**
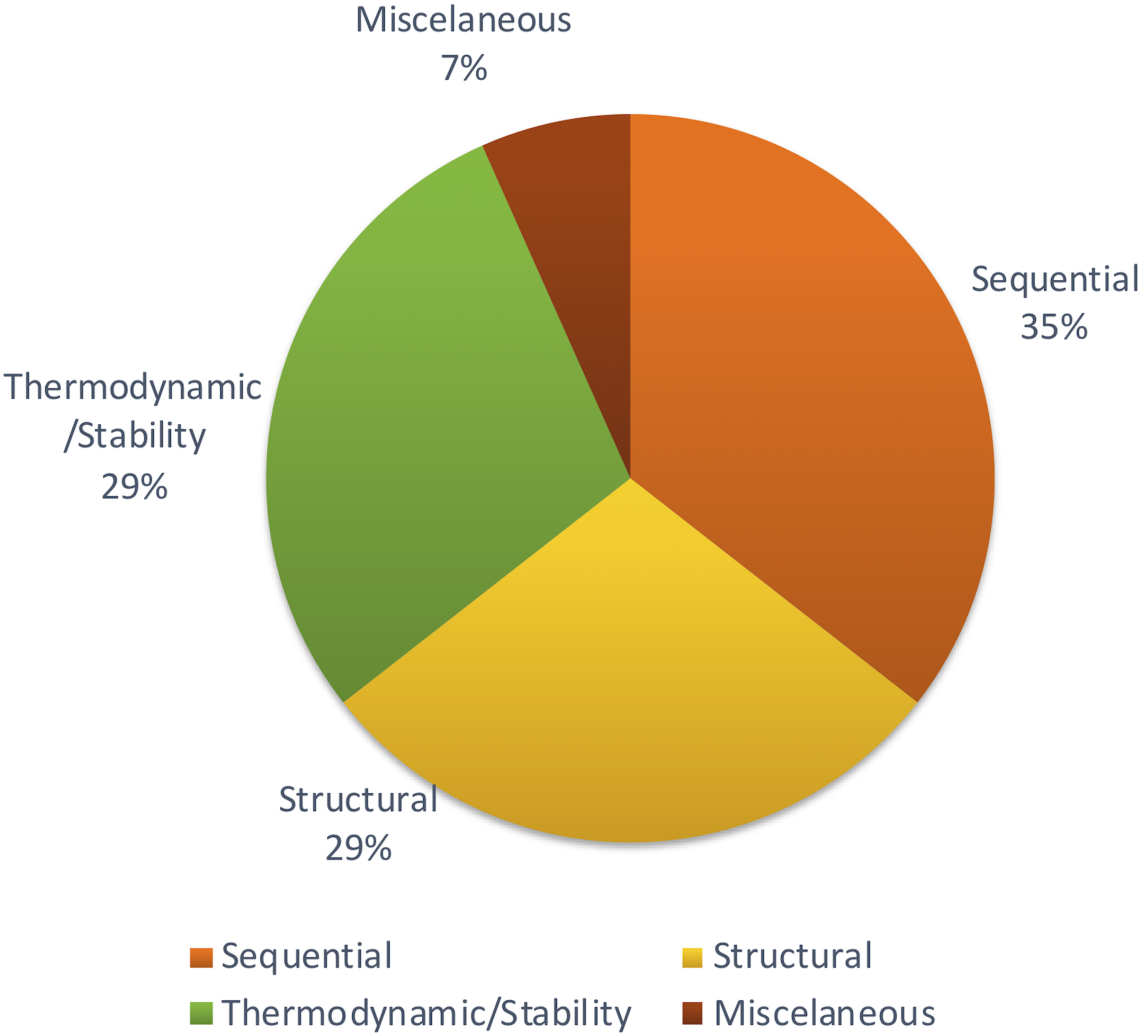
Types of features used.

Apart from those features, Xiao et al. (2011) focused on other methods to achieve structural features using network parameters. A few remaining studies haven’t described the feature set with adequate information. But most of the studies tend to follow the same set of features which were proven to be effective through previous studies. Figure 4 shows the distribution of types of features used in the primary studies.

Different studies have been conducted to show the impact of different sets of features. Some methods show that thermodynamic features (Yao et al., 2016) are better while another reports that sequential features (Yousef et al., 2016) are better. However, there is no concrete answer or common theme since there aren’t many studies comparing different feature types for plant miRNA prediction.

Whilst most studies utilized features extracted from data generated from various plant species, a few did use features extracted from non-plant species and then used this data to test their models’ performance on other species. Both Guan et al. (2011) and Kadri, Hinman & Benos (2009) used human miRNA data to train their models and then tested model performance on several plant species including the model plant species *Arabidopsis thaliana* as well as *Oryza sativa*. Both methods performed well on these species, with (Kadri, Hinman & Benos, 2009) achieving 97.4% and 85.7% of correctly predicted miRNA for *A.thaliana* and *O.sativa* respectively. Guan et al. (2011) was able to achieve 96.53% accuracy for *A.thaliana* and 97.61% for *O.sativa* as well an impressive 100% accuracy for *Chlamydomonas reinhardtii*. Similarly, (Vitsios et al., 2017) demonstrated an accuracy of between 90.7% and 82.9% for the identification of plant miRNAs using a model trained on animals. Xiao et al. (2011) was also able to achieve similar results in the detection of miRNA precursors trained on animal data, demonstrating an accuracy of 97.6% for plant data. The success of these studies indicates that plant and animal miRNAs do share some conserved sequence and structural characteristics.

The studies considered in this review all used machine learning algorithms to identify novel miRNAs in plant species. The selected primary studies used the following machine learning algorithms in their methods.

• Support Vector Machine (SVM) (Kecman, 2005)
• Random Forest (Breiman, 2001)
• Naive Bayes (Runkler, 2012)
• Decision Tree (Swain & Hauska, 1977)
• Hierarchical Hidden Markov Model (HHMM) (Fine, Singer & Tishby, 1998)
• ADABoost (Freund & Schapire, 1999)

Out of the above algorithms, 11 studies used SVM for their model. In general, models using SVMs have provided good overall performances in miRNA identification. Three other studies used Random Forest algorithm. The machine learning algorithms used are limited to the above list in the past decade.

**Figure 5:**
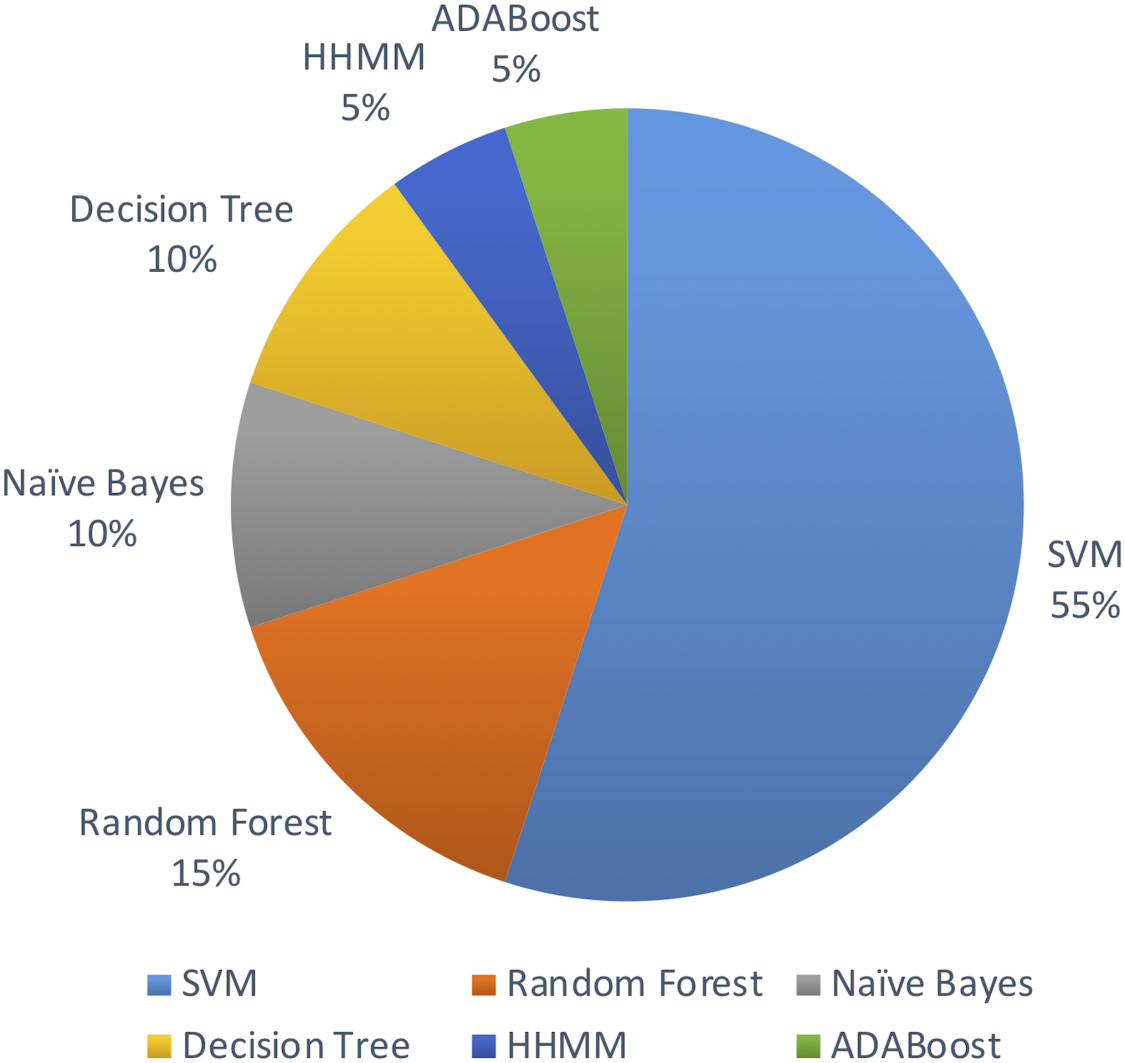
Machine learning algorithms used.

Figure 5 shows the distribution of machine leaning algorithms used in the past decade on identifying novel miRNAs in plants.

The inputs to these machine learning models consist of either pre-miRNA, mature miRNA or small RNA sequences. Meng et al. (2014) used both pre-miRNA and mature miRNA as the inputs to develop an integrated model for both miRNA and pre-miRNA prediction. Methods such as (Tseng et al., 2018; Douglass et al., 2016; Breakfield et al., 2012) used small-RNA sequencing data for their models. These methods still output the predicted miRNAs.

(Q3) How accurate and reliable are the developed models?

Considering the overall results reported by the authors, almost all the methods performed well in identifying novel plant miRNAs –many of them achieved very good accuracy values. Most of the studies used accuracy, recall, sensitivity and specificity to illustrate the performance of the model. Eleven studies used accuracy as a performance measure and 10 of those studies achieved accuracies above 90%. Even though the reported performances are not directly comparable, the highest accuracy of 99.48% was reported by Yousef et al., (2016). Considering the results presented by each study, all of them performed well and therefore, are seemingly reliable. All of the plant only methods perform well with accuracy values of above 90%. These performance values are based on the considered specific plant species and may not work for any species. Also, there is a potential for improving the performances by considering feature selection and advanced machines learning techniques. Note that the analysis presented here is only based on the performances reported by the authors.

While it may look like that many models are performing very well with performance values above 90%, we would like to highlight the fact that more than 90% of the models are developed/ tested with/on plant species with relatively less complex genomes such as A. thaliana (see Fig. 3). Therefore, we raise the concern that these models may not work for more complex plant genomes such as Wheat. With the recent sequencing of the whole wheat genome, identifying novel miRNAs and their functions is of utmost importance. But none of the existing methods reviewed in this survey focuses on complex plant species. The lack of high-quality plant data in popular knowledgebases such as miRBase (Kozomara, Birgaoanu & Griffiths-Jones, 2019) (which leads to lack of adequate training data) may be hindering the bioinformatics community from developing plant-based models for complex plant genomes.

(Q4) What kind of computational and/or experimental validation methods were used? How appropriate are those validation methods?

Except for two studies, all the other studies used a cross-validation technique for evaluating their machine learning models. Five-fold cross validation was used by eight studies while six studies used 10-fold cross validation. Using cross validation is helpful in performance evaluation of the developed models.

Experimental validation of putative novel miRNA’s is an important part of miRNA prediction. Of the 20 studies evaluated in this systematic review, only four (Tseng et al., 2018; Breakfield et al., 2012; Douglass et al., 2016; Sunkar et al., 2008) experimentally validated the presence of the novel miRNAs predicted by their machine learning methods. The most popular method was stem-loop PCR, employed by Tseng et al. (2018), Breakfield et al. (2012) and Douglass et al., 2016). Tseng et al. (2018) additionally utilized qPCR and (Sunkar et al., 2008) employed Northern blot analysis and small RNA blots. (Tseng et al., 2018) confirmed 18 out of 21 predicted miRNAs to be real miRNAs while (Sunkar et al., 2008) has tested and confirmed seven out of 13 predicted miRNAs. Breakfield et al. (2012) and Douglass et al. (2016) experimentally validated 8 of their predictions each to be true miRNAs.

(Q5) What are knowledge gaps, open problems and/or opportunities?

Computational miRNA identification is still a relatively young branch of biology and as such, it contains many knowledge gaps, open problems and opportunities. However, one of the most pressing is the need for the biological validation of computationally predicted miRNAs.

It’s become clear from studies conducted by Axtell & Meyers (2018), Taylor et al. (2014) and Taylor et al. (2017) that many of the miRNA sequences deposited in databases such as miRBase (Kozomara & Griffiths-Jones, 2014) are biologically implausible. Taylor et al. (2014) labeled one-third of all annotation plant miRNA loci and 75% of all plant miRNA families as questionable in miRBase release 20 (Kozomara & Griffiths-Jones, 2014). Similarly, (Axtell & Meyers, 2018) found that only 8.5% of land plant miRNA loci and 9.4% of land plant families are labeled as high confidence in miRBase version 21 (Kozomara & Griffiths-Jones, 2014).

Whilst there are many factors responsible for these observations, one of the causes may simply be developments in the understanding of miRNA biology. The last ten years have seen the release of two guidelines for the identification of plant miRNA identification, one of which was released in 2008 and the other in 2018 (Meyers et al., 2008; Axtell & Meyers, 2018). Prior to these releases, the first miRNA identification guide was produced in 2003 (Ambros et al., 2003). As all computational identification methods are based upon biological assumptions, it stands to reason that the use of tools that are based on inaccurate or out-of-date assumptions will yield biologically questionable results. Whilst this unmistakably calls for researchers to thoroughly inspect the methods of their chosen tools to discern the assumptions upon which it is based, this is not always a straightforward task. Most of the tools in this study made no reference to a specific guideline that was followed, which is of course not a necessity and in some cases would be inappropriate. The sources used may indeed be in accordance with the most recent guidelines or they may be expanding upon those guidelines, such as performed in Yousef, Allmer & Khalifa (2015), who investigated motif-based features for ab initio plant miRNA detection. Additionally, if there have been developments in the understanding of miRNA biology that have succeeded the information in the guidelines, it would, of course, make little sense to blindly abide by the guidelines. An additional complication is a lack of clarity in the methods. These tools are both biologically and computationally complex, and understanding the methods that underlie them may not be a straightforward task for experts of various domains. There is a need to ensure that the methods of such tools are written in such a way as to make clear the underlying assumptions. Failure to do so could lead to a tool being inappropriately selected, disregarded or improperly used. In some cases, this will require the user of such tools to read the proceeding studies that have been referenced in place of the method specifics.

Another cause of the questionable miRNA annotations that are deposited in databases is the unquestionable use of the databases themselves (Taylor et al., 2017). As discussed previously, many of the annotations within databases such as miRBase are questionable at best and at worst incorrect (Taylor et al., 2014; Taylor et al., 2017; Axtell & Meyers, 2018). As such, an additional opportunity for improvement presents itself to both computer scientists and biologists; the selection of high-confidence miRNA’s to be used as benchmarks. Of the papers discussed here all used either miRBase or its precursor the microRNA registry database, of which seven used miRBase version 20 or 21. Of these papers; (Yao et al., 2016; Yousef, Allmer & Khalifa, 2015; Yousef et al., 2016; Vitsios et al., 2017; Douglass et al., 2016; Tseng et al., 2018; Koh & Kim, 2017), only (Douglass et al., 2016) makes reference to the confidence of the sequences used. Whilst they do not explicitly say they used “high confidence” sequences, they specify they required either one or two types of experimental evidence dependant upon species and available evidence (Douglass et al., 2016). The addition of a “high confidence” tag was made available shortly after the release of miRBase version 20, and it allows users to “vote” if they agree with the “high confidence” tag or not (Kozomara & Griffiths-Jones, 2014). For studies that used miRBase prior to version 20, the use of experimentally-validated miRNAs shows that the miRNA sequences used were of high confidence. However only (Meng et al., 2014; Wu et al., 2011; Douglass et al., 2016) specify the use of experimentally validated sequences. Whilst utilizing only high-confidence miRNAs will increase the manual work required to obtain data from databases and will likely significantly decrease the number of available sequences which may reduce statistic power. However, it may be a necessity to reduce the rate at which false positive miRNAs are being deposited into databases. Whilst it may be outdated due to further miRBase updates, (Taylor et al., 2014) provides a link to a library of valid plant miRNAs in fasta format which can be utilized and/or built upon as a benchmark for future plant miRNAomes.

Another important factor in the influx of incorrect annotations is the unquestioning inclusion of all bioinformatically predicted miRNAs (Taylor et al., 2017). It is very likely that computational prediction programs will produce false positives, and the only way to avoid the inclusion of these incorrect annotations is the manual inspection of each positively identified miRNA against the most recent set of guidelines, such as those written by Axtell & Meyers (2018) and Taylor et al. (2017). Whilst this process will massively increase the manual requirements for miRNA identification, it will go some way in preventing the continuous influx of incorrectly annotation sequences into public databases (Taylor et al., 2017). However, the best form of verification of the biological presence of a miRNA is experimental validation. Of the papers discussed in this review, only four (Tseng et al., 2018; Douglass et al., 2016; Sunkar et al., 2008; Breakfield et al., 2012) incorporated some form of experimental validation. Of these, only two studies were based only upon the development of a miRNA prediction model or classifier (Tseng et al., 2018; Douglass et al., 2016). Both of these studies utilized small RNA-Seq data which may still yield false positive miRNA predictions and indeed, this is demonstrated by the experimental validation of predictions that used small RNA-seq data. For example, Tseng et al. (2018) experimentally confirmed the presence of only 18 out of 21 predicted novel miRNAs within two biological replicates and Douglass et al. (2016) was able to validate only two out of 12 high scoring putative miRNAs using their stringent criteria. Whilst it is likely that experimental validation will yield some level of false negative results, it may still be a necessity if progress is to made towards mapping the genuine miRNAome of a given species.

Due to the rising concern of poor miRNA annotations in databases, it is likely that many changes will be made by both database curators and researchers. For example, Axtell & Meyers (2018) recommend that all miRNAs identified through a homology-based approach only should be labeled as “putative”. In addition, the authors of miRBase (Kozomara & Griffiths-Jones, 2014) are aiming to incorporate a tiered confidence structure for miRNA entries as well as a text-mining based approach to categorize miRNA related articles and extract the biological meanings from the text. These changes may result in the alterations of miRNA annotations and as such, it may benefit biologists to utilize the miRBase change log function available from miRBase 22 or tools such as the miRBase Tracker (Van Peer et al., 2014; Kozomara & Griffiths-Jones, 2014). The use of these tools will aid biologists in understanding the annotation history of a given miRNA, and perhaps, in the future provide information regarding changes in supporting evidence.

Machine learning and feature selection methods related issues also exist in this field. Different groups have used various techniques for selecting negative data without having performed a comprehensive study on the most appropriate technique. But since the quality of the negative data heavily impacts machine learning models, this should directly be addressed. Also, as mentioned before, many authors use features proven to be most effective for animals on models developed for plants without comprehensive evaluation. This likely impacts the performance due to the noticeable differences between plant and animal miRNA sequences (Yao et al., 2016; Douglass et al., 2016). On top of this, some models have not considered feature selection at all (Silla, De O Camargo-Brunetto & Binneck, 2010; Williams, Eyles & Weiller, 2012; Xiao et al., 2011 etc.).

As mentioned above, most of the methods haven’t conducted experimental validation of the novel miRNAs predicted by the computational models. In fact, only 4 methods have validated their findings (Breakfield et al., 2012; Douglass et al., 2016; Tseng et al., 2018; Sunkar et al., 2008). Machine learning methods are not perfect; It is important to confirm if the predictions of the model are accurate in order to claim the finding of novel miRNAs. Also, the use of feature selection methods would be beneficial rather than using all available features for the model. But only some of the methods have used feature selection techniques. Considering the differences between plant and animal miRNA sequences, focusing on features specific to plants (instead of using the features that were found to work well for animal miRNAs), and identifying features effective for more complex genomes such as Wheat and Barley would be essential.

Use of other sophisticated machine learning algorithms would be beneficial in enhancing the performance of the tools. Apart from the machine learning algorithms mentioned in the primary studies, other opportunities are available with advanced models such as neural networks (Abe, 1997) and deep learning (LeCun, Bengio & Hinton, 2015). However, there needs to be a large dataset in order to use deep learning models and given the sparsity of experimentally validated sequences, this may not be an appropriate route at this time. As such, semi-supervised models that learns from both labeled and unlabeled examples may provide an added advantage. Due to the issues surrounding finding quality negative data, one-class classification or PU learning models (using Positive and Unlabeled samples) (Wu et al., 2017) may also be a fruitful choice.

## Conclusion

In this work, we have conducted a systematic review of ab initio plant miRNA identification tools that have been developed over the last decade. To achieve this, five questions were posed which aimed to elucidate the developments and assess the reliability and validity of the various methods used to identify novel plant miRNAs.

In total there are 20 studies that addressed plant miRNA identification using machine learning. Although it is a relatively small number of studies, most of the studies report promising results in the range of 90% of accuracy or above obtained through computational validation. Only 55% of the studies focused on only plants and even fewer of them focused on a specific plant species. This demonstrates a pressing need for plant specific and species specific methods. Compared with the dataset available for animal species, there is a relatively small number of experimentally verified plant miRNAs. This limits the authors and developers of machine learning tools, which require sometimes copious amounts of data for the training of their models. Recognizing the most informative features that are based on unique features of plant datasets will likely increase the accuracy of those methods. Whilst many studies continued using features from previous studies resulting in a large set of features, it’s important to verify that the assumptions that were made when the data was created are still in line with the present understanding of miRNA biology.

While it is true that the models are performing well, they are being tested on low quality data. So, we do raise this as a major concern. It is a well-known problem that a considerable number of predicted miRNAs are false predictions (Taylor et al., 2017). So, cleaning up the current knowledge bases should be a top priority. Otherwise, these errors will be propagated as well.

An additional challenge is that not all the developed software are accessible by the public. Some of them do not work as advertised due to technical issues and that further decreases the number of available methods with respect to plant miRNA prediction. Given that the intended audience of these tools would be biologists (i.e., non-experts in software development), extreme care must be taken in improving the availability, user friendliness and reliability. For the models involving different parameter options, guidelines must be provided in finding the optimum parameter values for the dataset of interest.

##  Supplemental Information

10.7717/peerj-cs.233/supp-1 Table S1 Categorization of the Primary Studies used in the Systematic Review

10.7717/peerj-cs.233/supp-2 Table S2 Publication Venues of the Primary Studies used in the Systematic Review

10.7717/peerj-cs.233/supp-3 Supplemental Information 1 PRISMA Checklist The PRISMA checklist contains standardized items with regards to steps that need to be taken when conducting a systematic review/meta-analysis. The rightmost column of this checklist contains the page and line numbers within the manuscript where each of those items is addressed.

## Additional Information and Declarations

### Data Availability

The following information was supplied regarding data availability: The raw data is available in the Supplemental File.
